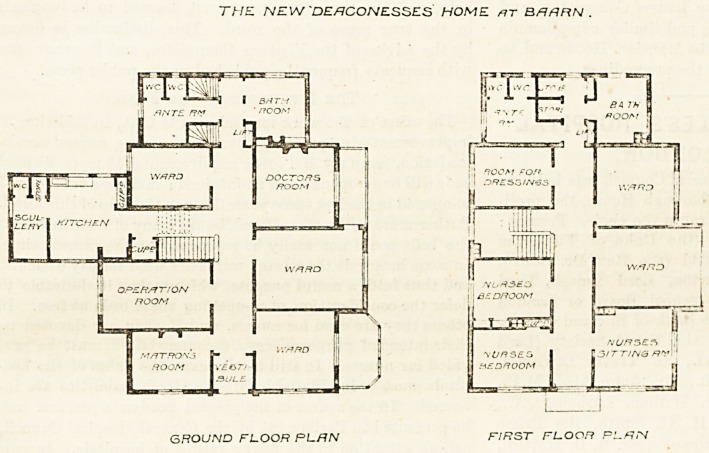# Hospital Construction

**Published:** 1900-02-17

**Authors:** 


### HOSPITAL CONSTRUCTION.

### THE NEW DEACONESSES HOME AT BAARN, HOLLAND.

THIS very much resembles some of our English cottage hospitals of the older type, built at a time when thorough cross-ventilation of the wards and efficient sanitary arrangements were not so strongly insisted on as they are at the present day. The ground plan is that of an irregular parallelogram, divided by a passage running east and west. On the south side lie the wards, two in number, and the surgeon's room. The surgeon's room is provided with an oblong bay, chiefly of glass, and a door from the adjoining ward opens into it. A similarly-constructed bay is placed in front of the ward at the south-west angle. On the north side

THE NEW DEACONESSES HOME AT BAARN.

GROUND FLOOR PLAN FIRST FLOOR PLAN

of the passage are the matron's room, the operation room, the main staircase, and another small ward. The operation room has a double window and a roof light. The floor is of cement and slopes slightly, so that the water runs off into the waste-pipes, and the room can be easily cleaned. The room is fitted up with all the appliances considered requisite by modern surgery. The kitchen projects northwards, and the bathroom and closets eastwards. The latter are not cut off by a a proper cross-ventilated passage as modern sanitary arrangements require, but there is a relatively large anteroom which will to a great extent obviate the drawbacks inherent to the plan adopted.

The first floor is similar to the ground floor, save that there is no room over the kitchen, nor, of course, over part of the operation-room. A nurses' sitting-room and two bedrooms are on this floor, and also two wards and a room "for dressings." The roof of the building is very high-pitched, and so gives rooms for two nurses, domestic servants, and storage. The wards have wide folding-doors, permitting patients to be carried out or in on stretchers. Apart from this admirable arrangement and the general compactness of the plan, there is no special feature in the hospital worthy of reproduction. Chimney-breasts are not shown in the wards, so we presume stoves are used for warming purposes, a method which should not be relied on unless joined with open fireplaces. The woodwork is of American pitch-pine, and is varnished, except the floors which are painted. The windows are furnished with shutters.

The plans are by Messrs. Aird and Wedderspon, of Gracechurch Street, assisted by L. C. Boissvan. The builder was M. Sweris, of Baarn. The home cost about 14,000 florins, or nearly £1,200. This would be considered cheap in England for the amount of accommodation obtained.

## Figures and Tables

**Figure f1:**